# Diverse evolutionary rates and gene duplication patterns among families of functional olfactory receptor genes in humans

**DOI:** 10.1371/journal.pone.0282575

**Published:** 2023-04-20

**Authors:** Yupeng Wang, Ying Sun, Paule V. Joseph

**Affiliations:** 1 BDX Research & Consulting LLC, Herndon, Virginia, United States of America; 2 Division of Intramural Research, National Institute on Alcohol Abuse and Alcoholism and National Institute of Nursing Research, National Institutes of Health, Bethesda, Maryland, United States of America; Institute of Parasitology and Biomedicine, SPAIN

## Abstract

In humans, odors are detected by ~400 functional olfactory receptor (OR) genes. The superfamily of functional OR genes can be further divided into tens of families. In large part, the OR genes have experienced extensive tandem duplications, which have led to gene gains and losses. However, whether different OR gene families have experienced distinct modes of gene duplication has yet to be reported. We conducted comparative genomic and evolutionary analyses for human functional OR genes. Based on analysis of human-mouse 1–1 orthologs, we found that human functional OR genes show higher-than-average evolutionary rates, and there are significant differences among families of functional OR genes. Via comparison with seven vertebrate outgroups, families of human functional OR genes show different extents of gene synteny conservation. Although the superfamily of human functional OR genes is enriched in tandem and proximal duplications, there are particular families which are enriched in segmental duplications. These findings suggest that human functional OR genes may be governed by different evolutionary mechanisms and that large-scale gene duplications have contributed to the early evolution of human functional OR genes.

## Introduction

Humans perceive a variety of odors. These odorants are detected by olfactory receptors (ORs), a type of seven-transmembrane domain G protein-coupled receptors [1–3]. The OR genes are one of the largest families in the human genome, consisting of ~400 protein-coding genes and another ~400 pseudogenes [4–7]. The OR genes can be further classified into tens of families and hundreds of subfamilies [4–6]. It is presumed that each subfamily of functional OR genes is dedicated to the detection of a particular class of odorants, though this does not preclude the recognition of a particular class of odorants by different subfamilies [4].

OR genes are distributed at many loci across 21 chromosomes in the human genome. Most OR subfamilies reside in a single chromosomal locus, and many loci encode only one or a few subfamilies. This pattern of chromosomal distribution was presumed to be caused by extensive tandem gene duplications among OR genes [8,9]. However, there are also a substantial number of cases in which OR genes of the same subfamilies are located on different chromosomal regions, and OR genes of different subfamilies are sometimes located closely in a chromosomal region [4,6,9]. Comparison of different species shows that the number of OR genes vary extensively among different species, and many species have a substantial number of pseudogenes [10–12]. It has been suggested that OR genes have experienced extensive gains and losses during evolution, presumably to meet the specific environmental/dieting niche(s) of each species [10,12–16]. These previous studies collectively suggest the complexity in the evolutionary mechanisms of OR genes.

In addition to single-gene duplications, such as tandem duplication, large-scale gene duplications, such as whole-genome duplication (WGD) and segmental duplication, have occurred in vertebrate evolution [17,18]. Genes created by different modes have often experienced different patterns of evolutionary tempos and gene gains and losses [19–21]. Moreover, gene relocations frequently occur during evolution [22–24]. However, little is known regarding the contributions of different gene duplication modes, especially large-scale gene duplication, to the evolution of OR genes.

The aim of this analysis is to better understand the evolutionary mechanisms of human functional OR genes via a variety of comparative genomic and evolutionary analyses. We have excluded pseudogenes as they are not protein-coding (*i*.*e*., not related to human odor detection).

## Results

### Human functional OR genes are under a more relaxed purifying selection and are more affected by balancing selection

We compiled a list of 399 functional OR genes in the human genome (see Methods). These functional OR genes comprise 17 families. The sizes and members of the families of human functional OR genes are shown in S1 Table. The family size of the various human functional OR genes vary from 3 to 68 members. Detailed information of the human functional OR genes can be viewed in S2 Table.

**Fig 1:**
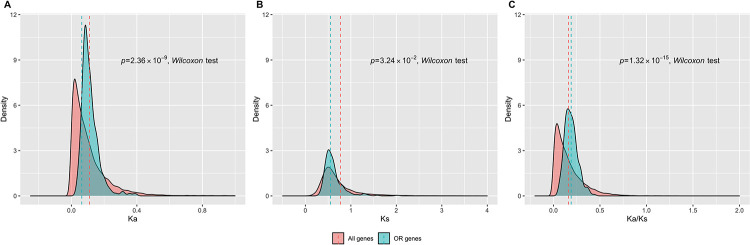
The human functional OR genes evolved relatively faster than the genome average. Comparison of Ka (A), Ks (B) and Ka/Ks (C) between human functional OR genes and the rest of the genes in the human genome**”**.

**Fig 2:**
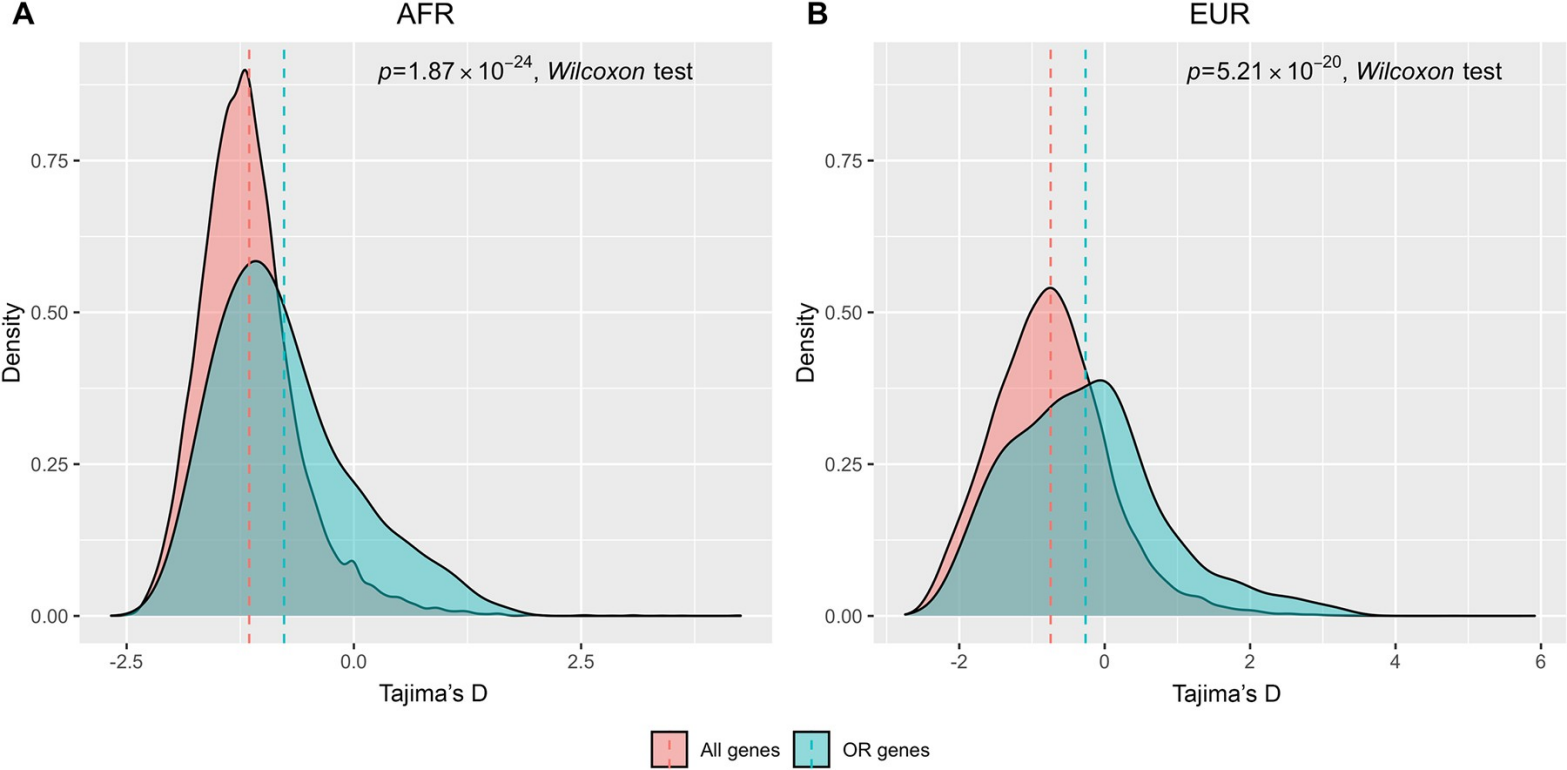
The human functional OR genes tend to have a higher Tajima’s D than the genome average. Comparisons of Tajima’s D between functional OR genes and all genes in the human genome. (A) In AFR. (B) In EUR.

We first studied the evolutionary rates of human functional OR genes based on analysis of human-mouse 1–1 orthologs. The evolutionary rates are denoted by non-synonymous substitution rate (Ka), synonymous substitution rate (Ks), and their ratio (Ka/Ks) (*i*.*e*., the rate of nonsynonymous substitutions corrected for neutral rates). Comparisons of Ka, Ks and Ka/Ks distributions between functional OR genes and all genes in humans indicate that functional OR genes evolved relatively faster in the human genome (Fig 1). As Ka/Ks is also indicative of selection pressure, a trend of higher Ka/Ks for functional OR genes indicates that human functional OR genes are, in general, under a relaxed purifying selection (Fig 1C). We next investigated the selection patterns of human functional OR genes in terms of deviation from neutrality. We computed Tajima’s D [25] for each functional OR gene based on the African (AFR) and European (EUR) populations of the 1000 Genomes Project [26]. Comparisons of Tajima’s D between functional OR genes and all genes in the human genome show that functional OR genes tend to have a higher Tajima’s D than the genome average (Fig 2), indicating that functional OR genes tend to be more affected by balancing selection in the human genome. Collectively, the above analyses suggest that in the human genome, functional OR genes tend to be under a more relaxed purifying selection and are more affected by balancing selection, further suggesting that human functional OR genes tend to preserve higher functional diversity, which may render a great variability in human olfactory cognition of numerous environmental olfactory stimuli.

### Human functional OR genes display diverse evolutionary rates among their families

**Fig 3:**
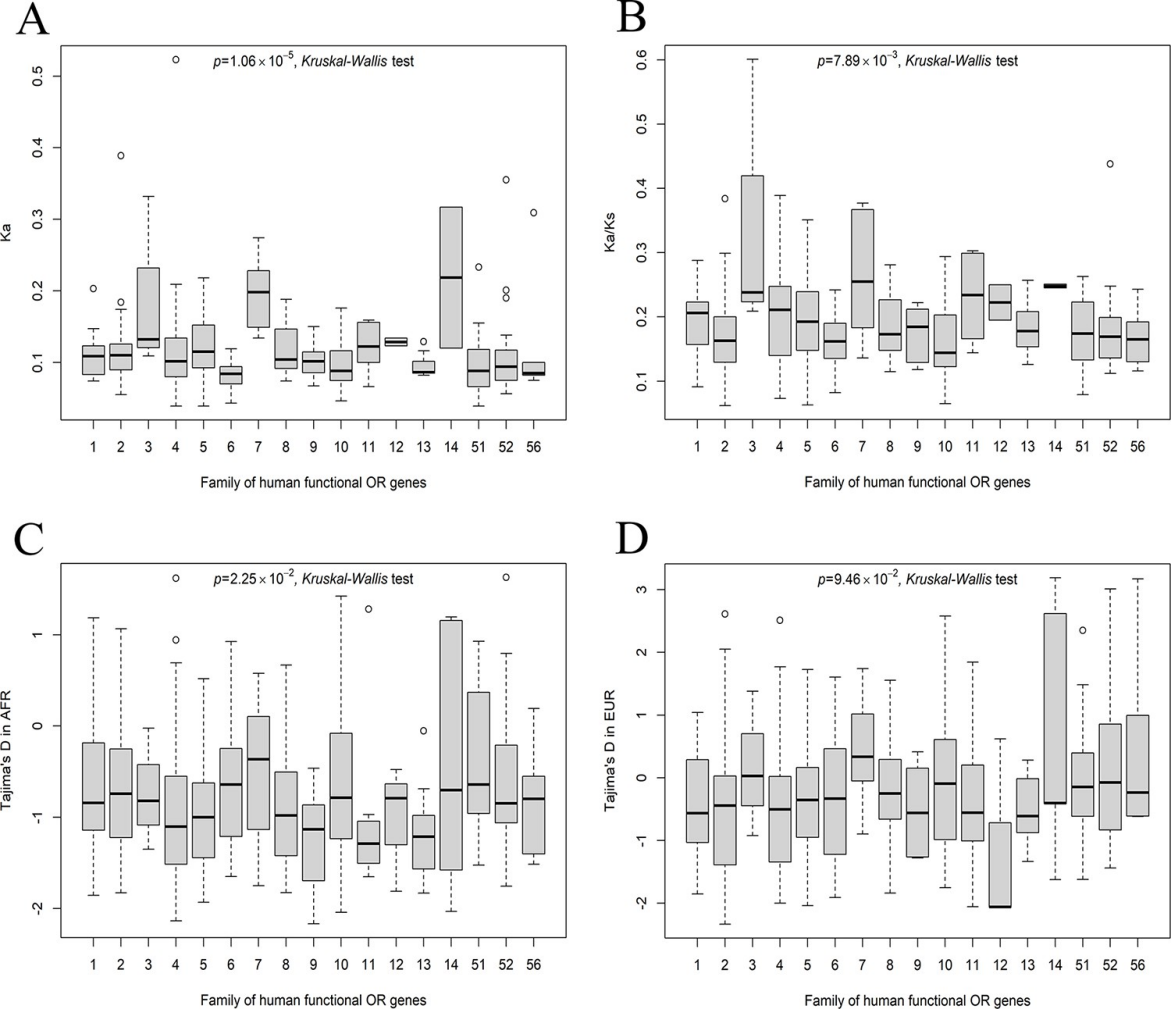
Different evolutionary rates and selection patterns among families of human functional OR genes. (A) Comparison of Ka. (B) Comparisons of Ka/Ks. (C) Comparisons of Tajima’s D in AFR. (D) Comparison of Tajima’s D in EUR.

We next investigated whether there are differences in the evolutionary rates among families of human functional OR genes. To this end, we compared Ka, Ka/Ks and Tajima’s D among families of human functional OR genes. Comparisons of Ka and Ka/Ks were statistically significant based on a *Kruskal–Wallis* test (*P* = 1.06×10^−5^ and 7.89×10^−3^, respectively) (Fig 3). We heuristically clustered families of human functional OR genes in terms of evolutionary rates and found that families 3, 7 and 14, whose sizes are relatively small (3, 11 and 6 members respectively) have a higher Ka (*P* = 1.42×10^−6^, *Wilcoxon* test) and Ka/Ks (*P* = 2.49×10^−4^, *Wilcoxon* test) than other families (Fig 3A and 3B). In contrast, the differences in Tajima’s D among families of human functional OR genes were much more moderate (*P* = 2.25 ×10^−2^ and 9.46 ×10^−2^ for AFR and EUR, respectively) (Fig 3C and 3D). These analyses suggest that families of human functional OR genes may differ in evolutionary rates, possibly pertaining to their different functions.

### Different gene synteny patterns among families of human functional OR genes

During the evolution of genomes, genes may remain on corresponding chromosomes (synteny) and corresponding orders (collinearity). It is known that in the human genome, most OR genes form local clusters, presumed to be created by extensive tandem duplications [8,9]. However, the extent of different functional OR genes remaining at ancestral chromosomal (*i*.*e*., syntenic) locations is poorly known. In addition, possible roles of synteny and collinearity among OR duplicated genes, presumed to be created by large-scale duplications, have been rarely reported.

**Fig 4:**
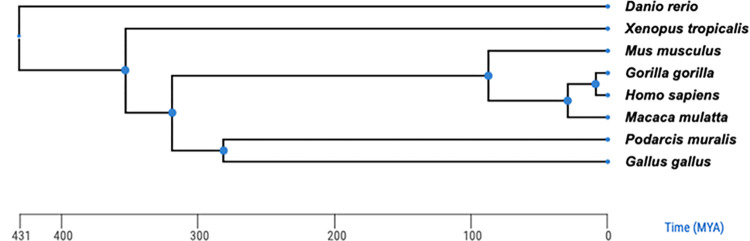
Evolutionary relationships between human and outgroup species. A dendrogram illustrating the evolutionary relationships of functional OR genes between the indicated species.

**Fig 5:**
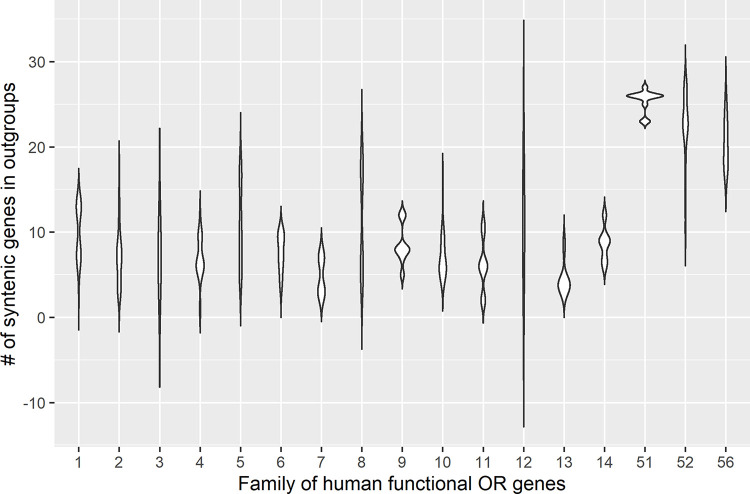
Families of human functional OR genes significantly differ in the number of syntenic genes in the outgroup species. Comparison of numbers of syntenic genes in outgroups species among families of human functional OR genes. The comparison was statistically significant (*P* = 1.42×10^−30^) based on *Kruskal–Wallis* test.

We performed both intra-species and cross-species synteny and collinearity analyses across eight species, including human, gorilla, macaque, mouse, chicken, lizard, frog and zebrafish. The evolutionary relationships of these species are shown in Fig 4. Genes with fewer syntenic genes across genomes are presumed to have been created more recently, or have undergone more relocations, often associated with the reshuffling of chromosomal segments. For each human functional OR gene, we computed the number of syntenic genes in the outgroup species. We found that families of human functional OR genes significantly differ in the number of syntenic genes in the outgroup species (Fig 5, *P* = 1.42×10^−30^, *Kruskal–Wallis* test). Notably, families 51, 52 and 56 show much higher numbers of syntenic genes in the outgroups than the other families (*P* = 6.22×10^−3^, *Wilcoxon* test), which may indicate that these families are more likely to perform conserved functions across the evolution of vertebrates.

Analysis of the intra-species collinear genes can indicate that duplicated genes have undergone large-scale gene duplication events, such as whole-genome duplication (WGD) or segmental duplication. We, thus, next generated the gene duplication modes among human functional OR genes (S3 Table), including 18 pairs of collinear (*i*.*e*. WGD/segmental) duplicates, and the data suggest that large-scale duplications may have substantially contributed to the evolution of human functional OR genes. To intuitively view gene duplication modes, we plotted WGD/segmental duplications and tandem duplications based on genome circles for all human functional OR genes and each family (S1 Fig). We computed enrichment of gene duplication modes for each family (S4 Table). Expectedly, the superfamily of human functional OR genes is enriched for tandem and proximal gene duplications. However, families 1, 14 and 52 are enriched for WGD/segmental duplications. This analysis suggests that large-scale duplications have played an essential role in the expansion and retention of human functional OR genes, especially in a few families. We observed that the locations of duplicate genes created by WGD/segmental duplications often coincide with tandem duplications. As tandem duplications often arise by unequal crossing-over [27], this observation suggests that a possible mechanism for human functional OR gene expansion could be frequent unequal crossing-over following large-scale gene duplications. We found that the Ks (a proxy for gene duplication ages) values of OR collinear duplicate pairs are significantly higher than OR tandem duplicate pairs (*P* = 2.70×10^−8^, *Wilcoxon* test. The above analyses collectively suggest that large-scale gene duplications have played an essential role during the evolutionary of human functional OR genes.

## Discussion

We observed that OR gene families significantly differ in the number of syntenic genes in the outgroup species. One explanation is that for more recent OR gene families, they may tend to have fewer syntenic genes for each member. We understand that genome annotation quality may be different among different genomes and annotation versions. As we are comparing the number of syntenic genes among human functional OR families, all OR families should be affected by similar levels of uncertainties, which is not likely to cause invalidation of our observation.

Small-scale duplications, such as tandem and transposed duplications, often have a higher evaporating rate [20]. This is consistent with the fact that in the human genome there are a comparable number of OR pseudogenes to functional OR genes [10–12]. In addition to tandem duplications, small-scale gene duplications can be further distinguished by proximal duplications (*i*.*e*., near one another but separated by a few genes) and by dispersed duplications (*i*.*e*., neither adjacent to each other in the genome nor within homologous chromosome segments) [23,28]. Tandem duplication is likely to arise from unequal crossing-over while proximal duplication is seemingly caused by localized transposon activities [23,28]. Distant single gene transposition may explain part of the dispersed duplicates segments [23,28]. Considering that OR genes are enriched for tandem and proximal genes, unequal crossing-over could be a primary mechanism for OR gene expansion and possibly for a subsequent pseudogenization. In particular, large-scale gene duplications were important for the evolution of particular OR gene families. Unequal crossing-over following segmental duplications could be an important evolutionary mechanism for several OR gene families. As there are contrasting evolutionary patterns among taste receptor genes [29,30], we suggest that diverse evolutionary mechanisms are a common phenomenon for olfactory sensory genes.

In this study, Ka/Ks were compared at superfamily and family levels. In the future we will compute Ka/Ks for branches of phylogenetic trees of OR genes and their p-values/q-values, in order to determine whether positive or negative selection is experienced. We will also associate the adaptive roles of OR genes/subfamilies with duplication bursts.

## Conclusions

In this study, we carried out comparative genomic and evolutionary analyses for human functional OR genes, and we were able to differentiate the specific evolutionary patterns for the various families of human functional OR genes. We found that functional OR genes show higher-than-average evolutionary rates in the human genome, but there are significant differences among families. We found evidence that suggests that human functional OR genes are more likely to be affected by balancing selection, though they show no obvious differences among families. Human functional OR gene families, including 1, 14 and 52, are enriched for WGD/segmental duplications. This study suggests that human functional OR genes may be governed by different evolutionary mechanisms and that large-scale gene duplications have played an essential role for the expansion and the retention of human functional OR genes.

## Methods

### Functional OR genes and gene families in humans

We obtained the list of human functional OR genes from Ensembl (https://uswest.ensembl.org/index.html) by searching the human genome for the keywords “olfactory and receptor”, restricting the returned result within the “gene” category and then removing pseudogenes by a custom Perl program.

The description of OR genes from Ensembl followed a convention “olfactory receptor family X subfamily X member X”. Thus, families of human functional OR genes were generated according to their gene description. For example, family 1 of human functional OR genes contain those OR genes with the keyword “family 1” in their description and without the keyword “pseudogene”.

The protein sequences, coding sequences and coordinates of human functional OR genes were also retrieved from Ensembl.

### Detection of gene synteny and collinearity

Protein sequences, CDS sequences in FASTA format and gene positions of the human genomes and seven outgroup genomes, including gorilla, macaque, mouse, chicken, lizard, frog and zebrafish, were retrieved from Ensembl. Versions of genome assembly are listed in S5 Table. For any genes that had more than one transcript, only the longest transcript was included in the annotation.

To search for homology between human and outgroup genomes, we conducted an all-vs-all BLASTP for each outgroup genome against human, and human against each outgroup genome, respectively, with an e-value cut-off of e^-10^ and the top five hits kept in each target genome. We also performed BLASTP for the human genome against itself with the same parameter setting and kept the top five non-self-hits for each gene.

To identify syntenic blocks between genomes, we concatenated all the above inter-/intra-species m6 BLASTP outputs into a.blast file and concatenated all gene positions of different genomes into a.gff file. Then we searched the syntenic blocks and collinear gene pairs using the software MCScanX [31] with the following default parameters: a match score of 50, gap penalty of −1, E-value of e^−5^, maximum gap size between any two consecutive protein pairs of 25 and at least five consecutive proteins to define a syntenic region. Syntenic blocks within the human genome were also generated using MCScanX with the same parameter setting.

### Analysis of duplicate gene origins

Origins of genes of a genome can be classified as singletons, whole genome/segmental (*i*.*e*., collinear genes in collinear blocks), tandem (consecutive repeat), proximal (in nearby chromosomal region but not adjacent) or dispersed (other modes than segmental, tandem, and proximal) duplications, depending on their copy number and genomic distribution. We used duplicate_gene_classifier from MCScanX [31] to classify origins of duplicate genes into the five classes for the human genome.

To detect gene duplications and their modes for the families of human functional OR genes, we first performed MCScanX-transposed [32], a software package based on execution of MCScanX, for the human genome, with the other seven genomes as outgroups. The MCScanX-transposed output was directly used as input for the downstream analysis program, detect_dup_modes_for_a_family, to detect duplicate gene pairs of different modes for the superfamily and families of human functional OR genes respectively.

We used origin_enrichment_analysis from MCScanX to identify potential enrichment of duplicate gene origins based on the result of duplicate gene origins. The *P*-value was calculated based on the null hypothesis that there was no association between the members of a gene family and a particular gene duplication mode and was corrected with the total number of duplication modes for multiple comparisons (i.e., *Bonferroni* correction).

### Ka and Ks calculation

Human and mouse 1-to-1 orthologous genes were retrieved from the vertebrate homology database (http://www.informatics.jax.org/homology.shtml). More specifically, we searched the “Mouse/Human Orthology with Phenotype Annotations” dataset (https://www.informatics.jax.org/downloads/reports/HMD_HumanPhenotype.rpt) for 1–1 orthologs. Then the OR gene names in the mouse genome were manually converted to gene names in Ensembl using the cross-reference links on their MGI (i.e. Mouse Genome Informatics, https://www.informatics.jax.org/) page.

We generated Ka and Ks values for duplicate gene pairs and orthologous gene pairs using the Yang and Nielsen method [33], available in the yn00 module of the PAML package [34]. We excluded the gene pairs with Ka or Ks < 0 from analysis. The Ka/Ks ratio was calculated by retrieving the ‘omega’ value of the MLmatrix.

### Neutral mutation test

We collected all OR genes’ variant calls of Europeans (EUR) and Africans (AFR) for Human (GRCh38.p13) from 1000 Genomes Project (https://www.internationalgenome.org) [26]. Then Tajima’s D [25] was calculated at the population and gene levels using vcftools v0.1.13 [35].

### Circle plots

Circle plots displaying gene duplication modes were drawn using the Circos software [36].

### Statistical analysis

Nonparametric tests were used for significance analysis due to non-normal distributions for the compared metrics. Comparisons between human functional OR genes and all genes were conducted by a *Wilcoxon* test. Comparisons among families of human functional OR genes were conducted by a *Kruskal–Wallis* test (one-way ANOVA on ranks).

## Supporting information

S1 Fig Diverse patterns in collinear and tandem relationships for human functional OR genes and each family, respectively. (TIF)

S1 Table Sizes and members of the human functional OR gene families. (DOCX)

S2 Table Detailed information of the human functional OR genes. (XLSX)

S3 Table Collinear, tandem and proximal duplicate genes among the human functional OR genes. (XLSX)

S4 Table Enrichment of gene duplication modes for the human functional OR genes and each family. (XLSX)

S5 Table Version information on reference genomes. (DOCX)

## References

[1] Buck L, Axel R. A Novel Multigene Family May Encode Odorant Receptors—a Molecular-Basis for Odor Recognition. Cell. 1991;65(1):175–87. doi: 10.1016/0092-8674(91)90418-x WOS:A1991FF77300019. 1840504

[2] Mombaerts P. Molecular biology of odorant receptors in vertebrates. Annu Rev Neurosci. 1999;22:487–509. doi: 10.1146/annurev.neuro.22.1.487 WOS:000079267400018. 10202546

[3] Buck LB. The molecular architecture of odor and pheromone sensing in mammals. Cell. 2000;100(6):611–8. Epub 2000/04/13. doi: 10.1016/s0092-8674(00)80698-4 . 10761927

[4] Malnic B, Godfrey PA, Buck LB. The human olfactory receptor gene family. P Natl Acad Sci USA. 2004;101(8):2584–9. doi: 10.1073/pnas.0307882100 WOS:000220140400063. 14983052 PMC356993

[5] Olender T, Lancet D, Nebert DW. Update on the olfactory receptor (OR) gene superfamily. Hum Genomics. 2008;3(1):87–97. Epub 2009/01/09. doi: 10.1186/1479-7364-3-1-87 ; PubMed Central PMCID: PMC2752031. 19129093 PMC2752031

[6] Glusman G, Yanai I, Rubin I, Lancet D. The complete human olfactory subgenome. Genome Res. 2001;11(5):685–702. doi: 10.1101/gr.171001 WOS:000168501600009. 11337468

[7] Zozulya S, Echeverri F, Nguyen T. The human olfactory receptor repertoire. Genome Biol. 2001;2(6):RESEARCH0018. Epub 2001/06/26. doi: 10.1186/gb-2001-2-6-research0018 ; PubMed Central PMCID: PMC33394. 11423007 PMC33394

[8] Young JM, Friedman C, Williams EM, Ross JA, Tonnes-Priddy L, Trask BJ. Different evolutionary processes shaped the mouse and human olfactory receptor gene families. Hum Mol Genet. 2002;11(5):535–46. Epub 2002/03/05. doi: 10.1093/hmg/11.5.535 . 11875048

[9] Niimura Y, Nei M. Evolution of olfactory receptor genes in the human genome. Proc Natl Acad Sci U S A. 2003;100(21):12235–40. Epub 2003/09/26. doi: 10.1073/pnas.1635157100 ; PubMed Central PMCID: PMC218742. 14507991 PMC218742

[10] Niimura Y, Matsui A, Touhara K. Extreme expansion of the olfactory receptor gene repertoire in African elephants and evolutionary dynamics of orthologous gene groups in 13 placental mammals. Genome Res. 2014;24(9):1485–96. Epub 2014/07/24. doi: 10.1101/gr.169532.113 . 25053675 PMC4158756

[11] Go Y, Niimura Y. Similar numbers but different repertoires of olfactory receptor genes in humans and chimpanzees. Mol Biol Evol. 2008;25(9):1897–907. Epub 2008/06/20. doi: 10.1093/molbev/msn135 . 18562338

[12] Dong D, He G, Zhang S, Zhang Z. Evolution of olfactory receptor genes in primates dominated by birth-and-death process. Genome Biol Evol. 2009;1:258–64. Epub 2009/01/01. doi: 10.1093/gbe/evp026 . 20333195 PMC2817421

[13] Hughes GM, Boston ESM, Finarelli JA, Murphy WJ, Higgins DG, Teeling EC. The Birth and Death of Olfactory Receptor Gene Families in Mammalian Niche Adaptation. Mol Biol Evol. 2018;35(6):1390–406. Epub 2018/03/22. doi: 10.1093/molbev/msy028 ; PubMed Central PMCID: PMC5967467. 29562344 PMC5967467

[14] Hayden S, Bekaert M, Crider TA, Mariani S, Murphy WJ, Teeling EC. Ecological adaptation determines functional mammalian olfactory subgenomes. Genome Res. 2010;20(1):1–9. Epub 2009/12/03. doi: 10.1101/gr.099416.109 . 19952139 PMC2798820

[15] Hughes GM, Teeling EC, Higgins DG. Loss of olfactory receptor function in hominin evolution. PLoS One. 2014;9(1):e84714. Epub 2014/01/07. doi: 10.1371/journal.pone.0084714 . 24392153 PMC3879314

[16] Niimura Y, Nei M. Evolutionary dynamics of olfactory and other chemosensory receptor genes in vertebrates. J Hum Genet. 2006;51(6):505–17. Epub 2006/04/12. doi: 10.1007/s10038-006-0391-8 . 16607462 PMC1850483

[17] Dehal P, Boore JL. Two rounds of whole genome duplication in the ancestral vertebrate. PLoS Biol. 2005;3(10):e314. Epub 2005/09/01. doi: 10.1371/journal.pbio.0030314 ; PubMed Central PMCID: PMC1197285. 16128622 PMC1197285

[18] Acharya D, Ghosh TC. Global analysis of human duplicated genes reveals the relative importance of whole-genome duplicates originated in the early vertebrate evolution. BMC Genomics. 2016;17:71. Epub 2016/01/24. doi: 10.1186/s12864-016-2392-0 ; PubMed Central PMCID: PMC4724117. 26801093 PMC4724117

[19] Blomme T, Vandepoele K, De Bodt S, Simillion C, Maere S, Van de Peer Y. The gain and loss of genes during 600 million years of vertebrate evolution. Genome Biol. 2006;7(5):R43. Epub 2006/05/26. doi: 10.1186/gb-2006-7-5-r43 ; PubMed Central PMCID: PMC1779523. 16723033 PMC1779523

[20] Lynch M, Conery JS. The evolutionary fate and consequences of duplicate genes. Science. 2000;290(5494):1151–5. Epub 2000/11/10. doi: 10.1126/science.290.5494.1151 . 11073452

[21] Hakes L, Pinney JW, Lovell SC, Oliver SG, Robertson DL. All duplicates are not equal: the difference between small-scale and genome duplication. Genome Biol. 2007;8(10). ARTN R209. WOS:000252100900007. 10.1186/gb-2007-8-10-r209 PMC2246283 17916239

[22] Wang Y, Ficklin SP, Wang X, Feltus FA, Paterson AH. Large-Scale Gene Relocations following an Ancient Genome Triplication Associated with the Diversification of Core Eudicots. PLoS One. 2016;11(5):e0155637. Epub 2016/05/20. doi: 10.1371/journal.pone.0155637 ; PubMed Central PMCID: PMC4873151. 27195960 PMC4873151

[23] Wang YP, Wang XY, Tang HB, Tan X, Ficklin SP, Feltus FA, et al. Modes of Gene Duplication Contribute Differently to Genetic Novelty and Redundancy, but Show Parallels across Divergent Angiosperms. Plos One. 2011;6(12). ARTN e28150 doi: 10.1371/journal.pone.0028150 WOS:000298171400036. 22164235 PMC3229532

[24] Han MV, Hahn MW. Inferring the History of Interchromosomal Gene Transposition in Drosophila Using n-Dimensional Parsimony. Genetics. 2012;190(2):813–U62. doi: 10.1534/genetics.111.135947 WOS:000300621200038. 22095076 PMC3276645

[25] Tajima F. Statistical-Method for Testing the Neutral Mutation Hypothesis by DNA Polymorphism. Genetics. 1989;123(3):585–95. WOS:A1989AX26700018. doi: 10.1093/genetics/123.3.585 2513255 PMC1203831

[26] Genomes Project C, Auton A, Brooks LD, Durbin RM, Garrison EP, Kang HM, et al. A global reference for human genetic variation. Nature. 2015;526(7571):68–74. Epub 2015/10/04. doi: 10.1038/nature15393 ; PubMed Central PMCID: PMC4750478. 26432245 PMC4750478

[27] Freeling M. Bias in plant gene content following different sorts of duplication: tandem, whole-genome, segmental, or by transposition. Annu Rev Plant Biol. 2009;60:433–53. Epub 2009/07/07. doi: 10.1146/annurev.arplant.043008.092122 . 19575588

[28] Wang Y, Wang X, Paterson AH. Genome and gene duplications and gene expression divergence: a view from plants. Ann N Y Acad Sci. 2012;1256:1–14. Epub 2012/01/20. doi: 10.1111/j.1749-6632.2011.06384.x . 22257007

[29] Wang YP, Sun Y, Joseph PV. Contrasting Patterns of Gene Duplication, Relocation, and Selection Among Human Taste Genes. Evol Bioinform. 2021;17. Artn 11769343211035141 doi: 10.1177/11769343211035141 WOS:000677606000001. 34366662 PMC8312168

[30] Shi P, Zhang JZ. Contrasting modes of evolution between vertebrate sweet/umami receptor genes and bitter receptor genes. Molecular Biology and Evolution. 2006;23(2):292–300. doi: 10.1093/molbev/msj028 WOS:000234718800007. 16207936

[31] Wang Y, Tang H, Debarry JD, Tan X, Li J, Wang X, et al. MCScanX: a toolkit for detection and evolutionary analysis of gene synteny and collinearity. Nucleic Acids Res. 2012;40(7):e49. Epub 2012/01/06. doi: 10.1093/nar/gkr1293 . 22217600 PMC3326336

[32] Wang Y, Li J, Paterson AH. MCScanX-transposed: detecting transposed gene duplications based on multiple colinearity scans. Bioinformatics. 2013;29(11):1458–60. Epub 2013/03/30. doi: 10.1093/bioinformatics/btt150 . 23539305

[33] Yang Z, Nielsen R. Estimating synonymous and nonsynonymous substitution rates under realistic evolutionary models. Mol Biol Evol. 2000;17(1):32–43. Epub 2000/02/10. doi: 10.1093/oxfordjournals.molbev.a026236 . 10666704

[34] Yang Z. PAML 4: phylogenetic analysis by maximum likelihood. Mol Biol Evol. 2007;24(8):1586–91. Epub 2007/05/08. doi: 10.1093/molbev/msm088 . 17483113

[35] Danecek P, Auton A, Abecasis G, Albers CA, Banks E, DePristo MA, et al. The variant call format and VCFtools. Bioinformatics. 2011;27(15):2156–8. Epub 2011/06/10. doi: 10.1093/bioinformatics/btr330 . 21653522 PMC3137218

[36] Krzywinski M, Schein J, Birol I, Connors J, Gascoyne R, Horsman D, et al. Circos: an information aesthetic for comparative genomics. Genome Res. 2009;19(9):1639–45. Epub 2009/06/23. doi: 10.1101/gr.092759.109 . 19541911 PMC2752132

