# Supplementary figures and images for: Diverse evolutionary rates and gene duplication patterns among families of functional olfactory receptor genes in humans
Source: PLoS One. 2023 Apr 20;18(4):e0282575. doi: 10.1371/journal.pone.0282575 (PMC10118112; doi:10.1371/journal.pone.0282575)

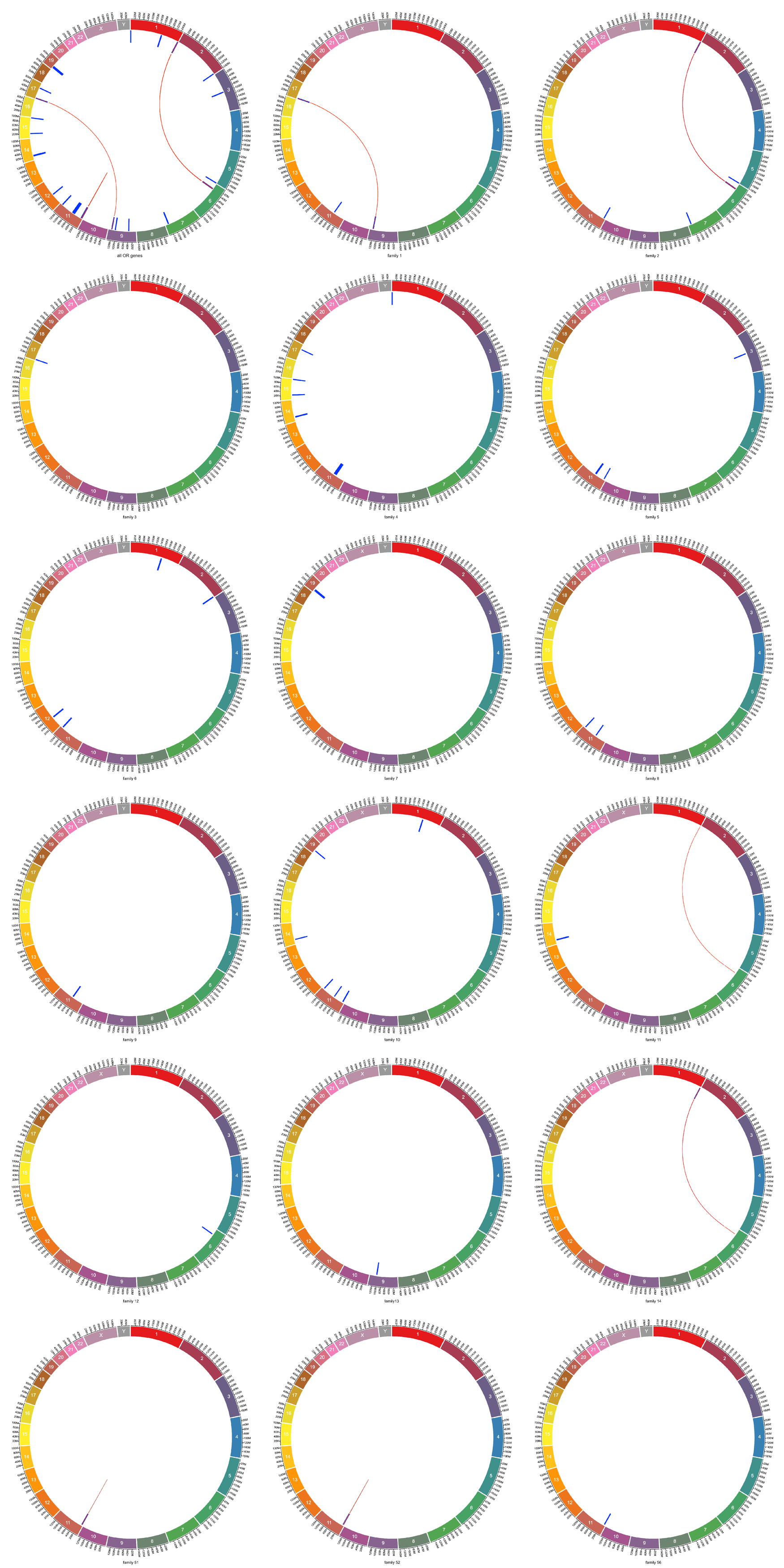

Supplement: S1 Fig — (TIF) [file pone.0282575.s001.tif]
