# Supplementary material for: Diverse evolutionary rates and gene duplication patterns among families of functional olfactory receptor genes in humans
Source: PLoS One. 2023 Apr 20;18(4):e0282575. doi: 10.1371/journal.pone.0282575 (PMC10118112; doi:10.1371/journal.pone.0282575)
Supplement: S1 Table — (DOCX) [file pone.0282575.s002.docx]

**S1 Table. Size and members for families of human functional OR genes**

| **Subfamily ID** | **Size** | **Members** |
| --- | --- | --- |
| 1 | 27 | OR1A2, OR1B1, OR1C1, OR1D2, OR1D5, OR1E1, OR1F1, OR1G1, OR1I1, OR1J1, OR1J2, OR1J4, OR1K1, OR1L1, OR1L3, OR1L8, OR1M1, OR1N2, OR1P1, OR1Q1, OR1S2, OR1A1, OR1E2, OR1L4, OR1L6, OR1N1, OR1S1 |
| 2 | 68 | OR2A2, OR2A5, OR2A7, OR2A12, OR2A14, OR2A25, OR2AE1, OR2AJ1, OR2AT4, OR2B2, OR2B3, OR2B6, OR2B11, OR2C1, OR2C3, OR2D2, OR2F2, OR2G2, OR2G3, OR2G6, OR2J1, OR2J2, OR2J3, OR2K2, OR2L3, OR2L5, OR2L8, OR2M2, OR2M3, OR2M4, OR2M5, OR2M7, OR2S2, OR2T1, OR2T2, OR2T3, OR2T5, OR2T6, OR2T7, OR2T8, OR2T10, OR2T11, OR2T12, OR2T27, OR2T29, OR2T33, OR2T34, OR2T35, OR2V1, OR2V2, OR2W1, OR2A1, OR2A4, OR2A42, OR2AG1, OR2AG2, OR2AK2, OR2AP1, OR2D3, OR2F1, OR2H1, OR2H2, OR2L2, OR2L13, OR2T4, OR2W3, OR2Y1, OR2Z1 |
| 3 | 3 | OR3A1, OR3A2, OR3A3 |
| 4 | 55 | OR4A5, OR4A8, OR4A15, OR4A16, OR4A47, OR4C3, OR4C5, OR4C6, OR4C13, OR4C16, OR4C45, OR4C46, OR4D1, OR4D5, OR4D6, OR4D11, OR4E1, OR4E2, OR4F4, OR4F5, OR4F6, OR4F15, OR4F17, OR4K1, OR4K2, OR4K3, OR4K5, OR4K13, OR4K14, OR4K17, OR4L1, OR4M1, OR4M2, OR4N5, OR4P4, OR4Q2, OR4Q3, OR4S1, OR4S2, OR4X1, OR4X2, OR4B1, OR4C11, OR4C12, OR4C15, OR4D2, OR4D9, OR4D10, OR4F3, OR4F16, OR4F21, OR4F29, OR4K15, OR4N2, OR4N4 |
| 5 | 51 | OR5A1, OR5A2, OR5AC1, OR5AC2, OR5AK2, OR5AL1, OR5AP2, OR5AR1, OR5AS1, OR5AU1, OR5B2, OR5B3, OR5B17, OR5B21, OR5C1, OR5D13, OR5D14, OR5D16, OR5D18, OR5F1, OR5G3, OR5H1, OR5H2, OR5H6, OR5H8, OR5H15, OR5I1, OR5J2, OR5K2, OR5K3, OR5K4, OR5L1, OR5L2, OR5M8, OR5M9, OR5P2, OR5R1, OR5T1, OR5T2, OR5T3, OR5W2, OR5AN1, OR5B12, OR5H14, OR5K1, OR5M1, OR5M3, OR5M10, OR5M11, OR5P3, OR5V1 |
| 6 | 30 | OR6A2, OR6B1, OR6B3, OR6C4, OR6C65, OR6C68, OR6C70, OR6C74, OR6C75, OR6F1, OR6J1, OR6K2, OR6K3, OR6K6, OR6M1, OR6N1, OR6N2, OR6P1, OR6Q1, OR6S1, OR6T1, OR6V1, OR6X1, OR6Y1, OR6B2, OR6C1, OR6C2, OR6C3, OR6C6, OR6C76 |
| 7 | 11 | OR7A5, OR7A10, OR7A17, OR7C1, OR7C2, OR7D2, OR7D4, OR7E24, OR7G1, OR7G2, OR7G3 |
| 8 | 23 | OR8B4, OR8B8, OR8D4, OR8G1, OR8G5, OR8H1, OR8H2, OR8H3, OR8I2, OR8J1, OR8J2, OR8K1, OR8K3, OR8K5, OR8S1, OR8U1, OR8A1, OR8B2, OR8B3, OR8B12, OR8D1, OR8D2, OR8J3 |
| 9 | 9 | OR9A2, OR9A4, OR9G1, OR9Q1, OR9Q2, OR9G4, OR9I1, OR9K2 |
| 10 | 38 | OR10A2, OR10A4, OR10A5, OR10A6, OR10A7, OR10AC1, OR10AG1, OR10C1, OR10D3, OR10G6, OR10G7, OR10G8, OR10H2, OR10H3, OR10H4, OR10H5, OR10J1, OR10J4, OR10J5, OR10K2, OR10P1, OR10Q1, OR10R2, OR10S1, OR10T2, OR10V1, OR10W1, OR10X1, OR10Z1, OR10A3, OR10AD1, OR10G2, OR10G3, OR10G4, OR10G9, OR10H1, OR10J3, OR10K1 |
| 11 | 9 | OR11A1, OR11G2, OR11H1, OR11H2, OR11H6, OR11H7, OR11H12, OR11L1, OR11H4 |
| 12 | 3 | OR12D1, OR12D2, OR12D3 |
| 13 | 13 | OR13A1, OR13C2, OR13C3, OR13C4, OR13C5, OR13C8, OR13C9, OR13D1, OR13F1, OR13G1, OR13H1, OR13J1, OR13C7 |
| 14 | 6 | OR14A2, OR14A16, OR14C36, OR14I1, OR14J1, OR14K1 |
| 51 | 22 | OR51A2, OR51A4, OR51A7, OR51B2, OR51B4, OR51B5, OR51D1, OR51E1, OR51E2, OR51F1, OR51F2, OR51G1, OR51G2, OR51I1, OR51I2, OR51L1, OR51M1, OR51S1, OR51T1, OR51V1, OR51B6, OR51Q1 |
| 52 | 26 | OR52B2, OR52B4, OR52B6, OR52D1, OR52E1, OR52E2, OR52E4, OR52E5, OR52E6, OR52H1, OR52I1, OR52I2, OR52J3, OR52K1, OR52K2, OR52L1, OR52M1, OR52N2, OR52N5, OR52W1, OR52A1, OR52A5, OR52E8, OR52N1, OR52N4, OR52R1 |
| 56 | 6 | OR56A1, OR56A4, OR56A5, OR56B4, OR56A3, OR56B1 |
