# Supplementary material for: Diverse evolutionary rates and gene duplication patterns among families of functional olfactory receptor genes in humans
Source: PLoS One. 2023 Apr 20;18(4):e0282575. doi: 10.1371/journal.pone.0282575 (PMC10118112; doi:10.1371/journal.pone.0282575)
Supplement: S5 Table — (DOCX) [file pone.0282575.s006.docx]

**S5 Table. Version information on reference genomes.**

| **Species** | **Genome name** | **Genome assembly** |
| --- | --- | --- |
| Human | Homo sapiens | GRCh38.p13 (GCA_000001405.28) |
| Gorilla | Gorilla gorilla | gorGor4 (GCA_000151905.3) |
| Macaque | Macaca mulatta | Mmul_10 (GCA_003339765.3) |
| Mouse | Mus musculus | GRCm38.p6 (GCA_000001635.8) |
| Chicken | Gallus gallus | GRCg6a (GCA_000002315.5) |
| Common wall lizard | Podarcis muralis | PodMur_1.0 (GCA_004329235.1) |
| Tropical clawed frog | Xenopus tropicalis | Xenopus_tropicalis_v9.1 (GCA_000004195.3) |
| Zebrafish | Danio rerio | GRCz11 (GCA_000002035.4) |
